# One-way or two-way sweet link between theobromine and depression?

**DOI:** 10.1186/s12888-023-04662-7

**Published:** 2023-06-07

**Authors:** Rafael Franco, Eva Martínez-Pinilla

**Affiliations:** 1 Molecular Neurobiology laboratory, Department of Biochemistry and Molecular Biomedicine, Faculty of Biology, Universitat de Barcelona, Diagonal 643, Prevosti Building, 08028 Barcelona, Spain; 2 CiberNed, Network Center for Neurodegenerative diseases, National Spanish Health Institute Carlos III, Madrid, Spain; 3 School of Chemistry, Universitat de Barcelona, Barcelona, Spain; 4 Department of Morphology and Cell Biology, Faculty of Medicine, University of Oviedo, Asturias, Spain; 5 Instituto de Neurociencias del Principado de Asturias (INEUROPA), Asturias, Spain; 6 Instituto de Investigación Sanitaria del Principado de Asturias (ISPA), Asturias, Spain

## Abstract

Theobromine is an abundant methylxanthine in cocoa/chocolate. A recent article in BMC Psychiatry concludes that theobromine consumption increases the risk of depression. In our opinion, it is difficult to make a correlation between dietary habits and the risk of depression, the diagnosis of which is not simple to make. Also, it is not easy to assess the amount of theobromine because it varies from one brand of chocolate to another and/or depending on the percentage of cocoa it has. Assuming that there is a correlation, we postulate that the conclusion may be the opposite, that is, that depressed individuals benefit from the intake of products containing theobromine. Since some antidepressant drugs alter the craving for sweet products, it would be interesting to try to correlate the data on theobromine intake with the kind of therapy used in depressed individuals.

## Commentary

Population-based studies are difficult to carry out, especially those related to dietary habits. Indeed, there are no individuals having the same diet and, therefore, it is very difficult to design the study and to collect the necessary information to obtain reliable conclusions.

The conclusion of a recently published cross-sectional study is: “*increased theobromine intake is associated with increased risk for depression*” [1]. Although the authors are cautious that more research is needed to confirm the finding, we were surprised because we have published articles of evidence that methylxanthines, including caffeine, theophylline, and theobromine, provide health benefits in some pathologies, neurodegenerative diseases among them, not only as potential disease-preventing agents but also as compounds that help in the daily life of patients (see, for instance, [2, 3]). Furthermore, the conclusion, in our opinion, is not well supported in the data presented or in the previous literature.

On the one hand, it is relevant to highlight the high number of participants in the study, 6903, and the huge workload to get data and to analyze them [1]. On the other hand, some of the details on the population heterogeneity are of concern and also the diversity of eating habits, which can vary substantially from one individual to another. For instance, the balance of depressed versus non-depressed changes with, among others, income and education. In addition, it seems that the main parameter in the study, namely the dietary intake of theobromine  (given in mg/day) was assessed just for the amount of compound taken the 24 h previous to a single interview. Even considering a more homogeneous population and that the age of depressed versus non-depressed would be equivalent, one wonders about the validity of the measure of theobromine consumption. There are many brands and types of products derived from cocoa, chocolate and others. Depending on the product, and therefore on the amount of cocoa and the cocoa component used in it, the final quantity of theobromine per gram can be quite different. Even if one really wants to know the precise number, it would be a real challenge to estimate the amount of theobromine ingested. By way of comparison, we know perfectly well that the amount of caffeine ingested is difficult to calculate even knowing the number of coffees and/or colas that have been taken. In colas it may be easier to guess, but the data on the quantity of caffeine in coffee can vary substantially from one variety of coffee to another, and also depending on the way the coffee is prepared and consumed, i.e., espresso and/or “American style”. If it is hard for caffeine, it is much harder for theobromine. We have checked two main sources of theobromine, dark chocolate and cocoa powder to mix with, for example, milk, and not a single number on theobromine content appears on the label. In summary: is it possible to have a good estimate of the amount of daily theobromine consumption? We doubt it, unless a careful study is undertaken; a recent one, uses high-performance liquid chromatography with diode-array detection, reports that depending on the commercial brand of chocolate milk, theobromine content goes from 1.7 to 12.2 mg/L [4].

Another concern is how depression is assessed. It is not easy to make an accurate diagnosis of depression, and we think it is reasonable to assume that there is no good way to measure “depression” in a population. It is doubtful that the method used in the article [1], that is, the resulting score considering those with more depressive symptoms versus those with fewer depressive symptoms can be considered a reliable parameter in correlation analyses. In the current edition of “*Diagnostic and Statistical Manual of Mental Disorders*” manual (DSM-5) [5], a diagnosis of depression requires having (i) a loss of interest or (ii) a low mood for 15 consecutive days. Nevertheless, to the best of our knowledge, there is no consensus on any depression score. Indeed, we wonder how an accurate analysis can be performed in a study in which “*presence of depression was defined as a score of 5 or above on the Patient Health Questionnaire*” [1]. Even if depression were a single entity and a score of 5 were given to depressed individuals, one wonders what the score might be for non-depressed people. This comment is not, however, written to discuss on the methodology but rather to try to gain insight from the data obtained.

As a matter of fact, there is neither a good way to score depression nor a good way to assess daily the theobromine intake. Is thus possible to make some optional or complementary conclusion based on the data reported by [1] and other data in the literature? Here, we propose an alternative view of the results of this recent article, which could be consistent with what is known about the consumption of cocoa/chocolate, the products with the highest theobromine content. The alternative view will not consider the correlation between theobromine intake with an increased risk of depression but with having depression. Suppose there is a real correlation between theobromine intake and depression. The key point here is whether theobromine consumption leads to low mood, decreased interest in performing daily activities or reduced actions for pleasure, or whether it is the other way around, people who are depressed are more likely to take food/beverages containing theobromine. The direct versus reverse causation related to chocolate and/or tea and/or coffee consumption has been previously considered. In 2009, a review based on the data presented in the conference “*Chocolate, Lifestyle, and Health*” (Milano, 2007) already confirmed that chocolate is something appreciated in our societies because it might be related to health issues [6]. Since then, the question has always revolved around whether there is a virtuous or vicious circle between chocolate and, say, affective disorders. A more recent systematic review (2017), indicated that the consumption of coffee, cocoa or tea could have protective effects against depression [7].

In a neurodegenerative disease such as non-familial Parkinson’s disease (PD), whose main risk factor is age, it is possible to assess the risk of suffering from the disease based on lifestyle and diet. There is strong data indicating that sustained intake of another methylxanthine, caffeine, decreases the risk of PD (see [3, 8] and references therein). Similarly, the consumption of beverages containing methylxanthines, tea and coffee, decreases the risk of suffering from another age-related disease, Alzheimer’s disease [9–11]. More difficult is to evaluate the risk of a disease that can occur at any age. In other words, no unidirectional link from methylxanthine consumption to disease can be established between an eating habit and a disease of contemporary course. Then, the option is to consider the two directional possibilities (i) theobromine consumption leads to low mood and decreased interest in performing daily activities, or (ii) people who are depressed and show reduced actions for pleasure are more likely to take food/beverages containing theobromine. We favor the second option based on previous data and on social behavior. Advanced societies assume that chocolate consumption reduces anxiety and/or improves happiness. In our country, Spain, we have always been addicted to hot chocolate, in its most classic version, hot chocolate with “churros”, a strip of fried dough. A relatively recent article has provided relevant information on the positive impact of chocolate consumption on mood and on the differential effects depending if chocolate is eaten in “*mindful consumption versus non-mindful consumption*” [12]. One of the conclusions of the work makes one wonder about the appeal of both theobromine and products that contain it: “*self-reported liking of the food partially mediated this effect. Chocolate appears to increase positive mood, but particularly when it is eaten mindfully*” [12].

At the mechanistic level, it must be noted that methylxanthines are mainly acting via blocking the four adenosine receptors, which are cell surface G protein-coupled receptors widely expressed in the brain. It is known that the effects of caffeine and theophylline are similar whereas the effect of theobromine is somewhat different. Methylxanthines are nonselective antagonists of adenosine receptors, and the potency of each of these compounds can vary from receptor to receptor. Theobromine may have another target, but the most likely hypothesis is that this compound affects adenosine receptors in a qualitatively and/or quantitatively different manner than caffeine and theophylline [13]. There is even a more convoluted possibility related to the craving of sweet that occurs when some therapeutic drugs are taken by patients. In fact, we think that information on specific therapies for depressed people would be very useful to detect differential effects of theobromine use depending on the drug administered.

Last but not least, chocolate often contains high concentrations of sugar and fat that can affect the mental state of the person who regularly consumes it. Cravings for sweet or sugary foods have been observed in people who are prone to depression or have periods of anxiety, something that seems to be related to increases in serotonin levels in the brain. This possibility would also fall into the category of reverse causation. Interestingly, a direct precursor of serotonin, tryptophan, is produced by the metabolism of some ingredients in cocoa/chocolate [14]. Other bioactive components of cocoa/chocolate are polyphenols, whose direct and/or indirect antioxidant role could be considered in a context of neuropsychiatric events. In this sense, a 2019 review compares, both qualitatively and quantitatively, the polyphenolic composition of coffee, tea, and cocoa [15], while a more recent review considers the evidence suggesting the potential of polyphenols in depression [16].

In summary, there are still many unknowns about the link between theobromine and depression, but certainly the answers would come from more research and new clinical trials that, while technically challenging, help distinguish the effects of theobromine and other components of cocoa. It would also be important to discover the exact correlation between chocolate intake and depression and the interaction of cocoa components with drugs used in the therapy of depressed patients.

## Data Availability

Not applicable.

## Authors’ information (optional)

**RF: December 31, 2022: >450 publications. >28,000 citations. Index h = 94 (Google Scholar)**.

orcid: 0000-0003-2549-4919.

scopus ID: 7202551094.

Researcher ID: C-3694-2015.

**EMP: December 31, 2022: 60 publications. >2,700 citations. Index h = 28 (Google Scholar)**.

orcid: 0000-0002-0572-0926.

scopus ID: 55745792500.

Researcher ID: C-7907-2013.
